# Epidemiology of malaria in the Taabo health and demographic surveillance system, south-central Côte d’Ivoire

**DOI:** 10.1186/s12936-015-1076-6

**Published:** 2016-01-06

**Authors:** Fidèle K. Bassa, Mamadou Ouattara, Kigbafori D. Silué, Lukas G. Adiossan, Nahoua Baikoro, Siaka Koné, Moussan N’Cho, Mahamadou Traoré, Bassirou Bonfoh, Jürg Utzinger, Eliézer K. N’Goran

**Affiliations:** Unité de Formation et de Recherche Biosciences, Université Félix Houphouët-Boigny, Abidjan, Côte d’Ivoire; Centre Suisse de Recherches Scientifiques en Côte d’Ivoire, Abidjan, Côte d’Ivoire; Hôpital Général de Taabo, Taabo-Cité, Côte d’Ivoire; Centre de Recherche et de Lutte contre le Paludisme, Institut National de la Santé Publique, Abidjan, Côte d’Ivoire; Department of Epidemiology and Public Health, Swiss Tropical and Public Health Institute, Basel, Switzerland; University of Basel, Basel, Switzerland

**Keywords:** Malaria, *Plasmodium*, Health and demographic surveillance system, Cross-sectional survey, Côte d’Ivoire

## Abstract

**Background:**

A deep understanding of the local epidemiology of malaria is essential for the design and implementation of setting-specific control and elimination efforts. In Côte d’Ivoire, new initiatives are underway to reduce the burden of malaria, which requires high-quality longitudinal data. The epidemiology of malaria was studied in the Taabo health and demographic surveillance system (HDSS) in south-central Côte d’Ivoire and implications for control are discussed.

**Methods:**

Two cross-sectional surveys were carried out in the rainy season of June/July in 2010 and 2011. Inhabitants of approximately 7 % of randomly selected households in the Taabo HDSS were invited to participate. People were clinically examined, ear temperature was measured and spleen size determined. Finger-prick blood samples were collected and subjected to a rapid diagnostic test (RDT). Additionally, thick and thin blood films were prepared on microscope slides and diagnosed under a microscope for *Plasmodium* infection and parasitaemia. Haemoglobin (Hb) level was determined using a HemoCue device.

**Results:**

A total of 1187 and 1264 people in 2010 and 2011, respectively, had complete data records. The prevalence of *Plasmodium* infection was 46.0 % in 2010 and 56.6 % in 2011, owing to a statistically significant difference (*p* < 0.05). Males showed a higher *Plasmodium* infection prevalence than females (49.6 and 62.8 % versus 42.6 and 51.2 %; respectively, in 2010 and 2011; both *p* < 0.05). The highest malaria prevalence was observed among infants and young children (aged ≤9 years). The risk of *Plasmodium* infection was significantly higher in villages compared to small hamlets and urban settings (*p* < 0.05). Fever, Hb level and splenomegaly were associated with parasitaemia.

**Conclusion:**

Malaria is highly endemic in the Taabo HDSS in south-central Côte d’Ivoire with considerable spatial heterogeneity of *Plasmodium* infection. There is a pressing need to scale-up control interventions against malaria.

## Background

Substantial progress has been made in the understanding of the epidemiology of malaria and ways to control or eliminate the disease. In particular, integrated approaches using vector control measures, e.g. long-lasting insecticidal nets (LLINs) and artemisinin-based combination therapy (ACT) considerably reduced the burden of malaria over the last decade [1, 2].

In Côte d’Ivoire, an over 10-year long sociopolitical crisis jeopardized malaria control. Nevertheless, different initiatives have been implemented by the national malaria control programme with financial support from the Global Fund to Fight AIDS, Tuberculosis and Malaria. However, a clear downward trend in the number of malaria cases has yet to be registered [3]. It must be noted that there is a paucity of accurate and coherent information about the local epidemiology of malaria. Most of the recent data stem from Abidjan, the economic capital of Côte d’Ivoire, usually obtained from health facilities [4–6]. Although health facility-based data can be helpful for assessing the malaria burden, the data must be used with full recognition of their limitations, since many people may not seek treatment in a health facility [7] and diagnostic-treatment algorithms are often empirical [8]. It follows that population-based data using accurate diagnostic methods are needed to provide precise estimates of the disease burden in different regions and contexts, as malaria control efforts are being intensified.

The aim of the present study was to assess the local epidemiology of malaria in the Taabo health and demographic surveillance system (HDSS) of south-central Côte d’Ivoire [9]. An in-depth analysis of 712 deaths recorded in the Taabo HDSS between 2009 and 2011 revealed that malaria was the most important cause of death (n = 129, 18.0 %) [10]. Here, results from two cross-sectional surveys conducted in the rainy season of 2010 and 2011 are reported.

## Methods

### Ethical considerations

The study protocol was approved by the institutional research commission of the Centre Suisse de Recherches Scientifiques en Côte d’Ivoire (CSRS; Abidjan, Côte d’Ivoire). Ethical approval was granted by the Comité National d’Ethique et de la Recherche (CNER) of Côte d’Ivoire (reference no. 1086 MSHP/CNER). Participation was voluntary and all study subjects provided written informed consent, with parents/legal guardians signing on behalf of children/adolescent below the age of 18 years.

### Study area

The study was conducted in the Taabo HDSS, located in the Taabo district, some 150 km north-west of Abidjan. The Taabo HDSS is situated in a forest-savannah transitional zone and has a surface of approximately 1000 km^2^. At the onset of operation in late 2008/early 2009, the population in the Taabo HDSS was 37,792 inhabitants. There is a small town (Taabo-Cité), 13 main villages and over 100 small hamlets. The climate is tropical, with two rainy seasons (March to July and October/November) and two dry seasons (December to February and August/September). Due to recent climate change, no clear seasonal delimitation is observed any longer and there is a tendency of just two seasons [11]. The average annual precipitation in the years 2010–2011 was 1284 mm, the mean annual temperature varied from 27.6 to 28.3 °C and the average relative humidity ranged between 76.6 and 79.6 % (data obtained from the nearby Lamto Station for Research in Geophysics and Ecology). People in the Taabo HDSS are mainly engaged in subsistence farming (yams, banana, maize and cassava). Cocoa and coffee are used as cash crops. There is also some livestock production (cattle, small ruminants, pigs and poultry) and fishery. A key environmental feature of the Taabo HDSS is the man-made Lake Taabo, impounded in the late 1970s after the construction of a large dam across the Bandama River, used for hydroelectric power production [12].

The Taabo HDSS has been launched in mid-2008 and serves as a platform for high-quality longitudinal data on all the residents, with particular emphasis on demography (pregnancy, birth, migration and death), health and socioeconomic status. Data are collected prospectively, usually pursuing three rounds per year to obtain key demographic data. Additionally, once every 2nd year, socioeconomic data are collected. The activities of the Taabo HDSS began by a baseline census in late 2008/early 2009, during which all the households of the district of Taabo were visited and georeferenced. Data were collected on household composition, house characteristics, household asset ownership, people’s ethnicity, nationality, religion, age, sex, education and employment.

From 2009 to 2011, community-based deworming against soil-transmitted helminthiasis (using albendazole or mebendazole) and schistosomiasis (using praziquantel) was undertaken in the whole area under surveillance under the leadership of district health personnel with support of Taabo HDSS staff. A series of specific research projects provided additional treatment to study participants found positive for specific parasitic infections [9, 13–15].

### Cross-sectional surveys

Two cross-sectional surveys were conducted; in 2010 and 2011, during the main rainy season at the end of June/early July. Study participants were invited for a finger-prick blood sample that was utilized for measuring haemoglobin (Hb) level, employing a HemoCue device (HemoCueB-Hemoglobin; Angholm, Sweden). Additionally, a rapid diagnostic test (RDT) was performed and thick and thin blood films were prepared on microscope slides for *Plasmodium* diagnosis. The RDT (ICT Diagnostics; Cape Town, South Africa) was used to detect *Plasmodium falciparum* histidine rich protein 2 (HRP 2). This RDT was shown to detect 86.9 % of test samples with wild-type *P.falciparum* at a concentration of 200 parasites/μl of blood and 98 % of test samples with a concentration of 2000 parasites/μl of blood, with 0 % of false positives in negative samples [16]. The results from the RDTs were used to guide treatment. Participants were also subjected to a clinical examination. Temperature was measured using a digital ear thermometer (Omron Gentle Temp 510; Kyoto, Japan). Spleen size was determined by a physician according to Hackett’s grading [17].

### Sampling technique

Study participants were selected from the readily available Taabo HDSS database. All members of approximately 7 % of the households were selected in each of the two cross-sectional surveys. The sampling design was based on clusters stratified according to residency (i.e. hamlet, village and Taabo-Cité) and the geographical location of residency relative to the Bandama River and Lake Taabo. Four strata were distinguished: (1) households situated in hamlets that were at least 5 km away from the river or lake; (2) households located in hamlets in close proximity to the river or lake (<5 km); (3) households in the main villages far from the river or lake; and (4) households in the main villages in close proximity to the river or lake and households from urban Taabo-Cité. The total number of individuals invited to participate in the years 2010 and 2011 was 2530 and 2621, respectively. Households were randomly selected in each stratum with a probability proportional to their population size.

### Laboratory procedures

Thick and thin blood films were transferred to the laboratory of the district hospital in Taabo-Cité and stained with 3 % Giemsa for 45 min. Parasitaemia was determined from thick blood films by counting the number of parasites per 200 white blood cells (WBC) or 500 WBC if less than 10 parasites had been detected, assuming a mean WBC count of 8000/μl of blood. A slide was classified as negative if no *Plasmodium* asexual form or gametocyte was found after counting 500 WBC. Thin blood films were examined for *Plasmodium* species identification, examining 100 microscopic fields. Quality control was performed by a senior technician, who was blinded to the results obtained from the first reader. Slides with conflicting results were re-read by a third microscopist and the results discussed among the technicians until consensus was reached.

### Statistical analysis

Demographic, clinical and laboratory data were double entered and validated in an Access 2007 database (Microsoft Corp., Redmond, USA). Fever was defined as an ear temperature ≥37.5 °C. Anaemia was defined according to guidelines put forth by the World Health Organization (WHO) [18]. In brief, children below the age of four years were considered anaemic if they had an Hb level <11.0 g/dl; children aged 5–11 years if they had an Hb level <11.5 g/dl; females aged 12-14 years who had an Hb level <12.0 g/dl; females aged ≥15 years with an Hb <12.0 g/dl; and males aged ≥13 years who had an Hb < 13.0 g/dl. To examine the effects of age on parasitaemia, age was stratified into six groups according to WHO guidelines [19]: <1, 1–4, 5–9, 10–14, 15–19 and ≥20 years. The same stratification was used for fever and spleen size. Individuals with missing or incomplete data were excluded from specific analysis that requested a given parameter under consideration.

Descriptive statistics were used to summarize demographic characteristics of the study sample. Comparisons between groups for categorical parameters were carried out by the Pearson’s χ^2^ test. Differences in geometric mean of parasitaemia, mean age and mean spleen size between groups were analysed by Wilcoxon test (two groups) or Kruskal–Wallis test (more than two groups) in case the Shapiro normality test was significant. Odds ratios (ORs) were estimated using logistic regression to compare the risk of *Plasmodium* infection between groups. For all analyses, difference was regarded as significant if the *p* value of the test statistic was <0.05. Data analyses were performed using R statistics (R Core Team, 2013).

## Results

### Demographic characteristics

A total of 1187 and 1264 individuals participated in the cross-sectional surveys conducted in 2010 and 2011, respectively. The number of people sampled, stratified by age group, sex and residency, is summarized in Table 1. In both cross-sectional surveys, most of the participants were young children and adolescents (58.7–58.9 %). The average age was 20.8 years (range 1 month–87 years, standard deviation (SD) = 18.8 years) in 2010 and 20.9 years (range 1 month–82 years, SD = 19.1 years) in 2011. There was no significant difference in the average age of participants in the two surveys.

**Table 1:** Demographic characteristics of the sample populations in the Taabo HDSS, stratified by year of survey

	2010		2011	
	N	%	N	%
All participants	1187	100	1264	100
Gender				
Male	575	48.4	588	46.5
Female	612	51.6	676	53.5
Age group (years)				
<1	46	3.9	49	3.9
1–4	179	15.1	189	14.9
5–9	245	20.6	273	21.6
10–14	165	13.9	181	14.3
15–19	62	5.2	52	4.1
≥20	490	41.3	520	41.2
Residency				
Hamlets	200	16.8	230	18.2
Villages	782	65.9	871	68.9
Urban	205	17.3	163	12.9

*N* Number of individuals

### *Plasmodium* infection

**Fig. 1:**
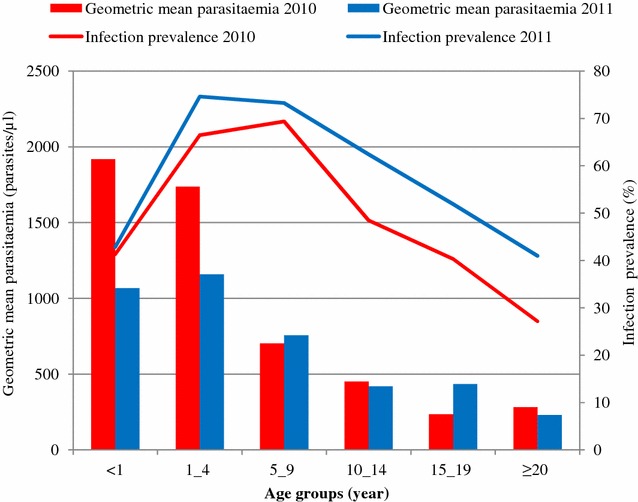
Age-specific prevalence of *Plasmodium* infection and geometric mean parasitaemia of the study population in the Taabo health and demographic surveillance system, south-central Côte d’Ivoire, stratified by year of survey

Table 2 summarizes the prevalence of *Plasmodium* infection, stratified by year of survey, participants’ residency and sex. The overall prevalence of *Plasmodium* infection in 2011 was significantly higher than in 2010 (46.0 versus 56.6 %; *p* < 0.01). Males showed a significantly higher prevalence of *Plasmodium* infection than females (49.6 and 62.8 versus 42.6 and 51.2 %; respectively, in 2010 and 2011; both *p* < 0.05). The prevalence of *Plasmodium* infection was significantly higher among individuals living in villages compared to residents from small hamlets or urban settings (*p* < 0.001). *Plasmodium* infection occurred in all age groups. The highest prevalence of infection was observed in infants and young children (age ≤9 years). Prevalence decreased with age (Fig. 1).

**Table 2:** Prevalence of *Plasmodium* infection and geometric mean parasitaemia of the sample population in the Taabo HDSS

	Prevalence of *Plasmodium* infection				GMP (parasites/µl of blood)	
	2010 (%)	OR (95 % CI)	2011 (%)	OR (95 % CI)	2010	2011
Overall	46.0^a^		56.6^b^		632.5	520.6
Gender						
Male	49.6 (285/575)	1.00	62.8 (369/588)	1.00	617.9	479.1
Female	42.6 (261/612)	0.8 (0.60–0.95)^†^	51.2 (346/676)	0.6 (0.49–0.77)^†^	648.8	568.7
*p*-value	<0.05		<0.001		0.822	0.177
Residency						
Hamlets	40.0 (80/200)	1.00	49.1 (113/230)	1.00	678	401.7
Villages	50.9 (398/782)	1.6 (1.13–2.13)^†^	60.5 (527/871)	1.6 (1.18–2.12)^†^	611.7	586.5^a^
Urban	33.2 (68/205)	0.7 (0.49–1.11)	46.0 (75/163)	0.9 (0.59–1.31)	708.8	332.8^b^
*p*-value	<0.001		<0.001		0.856	0.006

For each parameter, different letters in the same row or column indicate that their difference is significant (*p* < 0.05)

*CI* confidence interval, *GMP* geometric mean parasitaemia, *OR* odds ratio

^†^ Notes that *p*-value < 0.05

### *Plasmodium* species-specific prevalence rates

Three human malaria species were detected in the study area: *P. falciparum*, *Plasmodium malariae* and *Plasmodium ovale*, with the former representing the predominant species (98.9 and 99.7 % of all positive thick and thin blood films examined in 2010 and 2011, respectively). *Plasmodium malariae* accounted for 1.1 and 0.2 % of the infections, respectively, while *P.ovale* was rarely observed (one mixed infection with *P. falciparum* in 2011).

### *Plasmodium* parasitaemia

Most infections were of low to moderate parasitaemia (16-9999 parasites/μl of blood). The proportion of infections with high parasitaemia (≥10,000 parasites/μl of blood) was 8.6 % in 2010 and 5.2 % in 2011. The geometric mean parasitaemia of positive samples was similar in both years (Table 2). The geometric mean parasitaemia was higher in the youngest age groups, compared to older counterparts (Fig. 1). With regard to sex, the difference in the geometric mean parasitaemia showed no consistent difference. With regard to parasitaemia and participants’ residency, there was no statistically significant difference in 2010. However, in 2011, parasitaemia was significantly higher in village residents compared to people living in small hamlets or the urban setting (Table 2).

### Fever prevalence and relation to *Plasmodium* infection

**Fig. 2:**
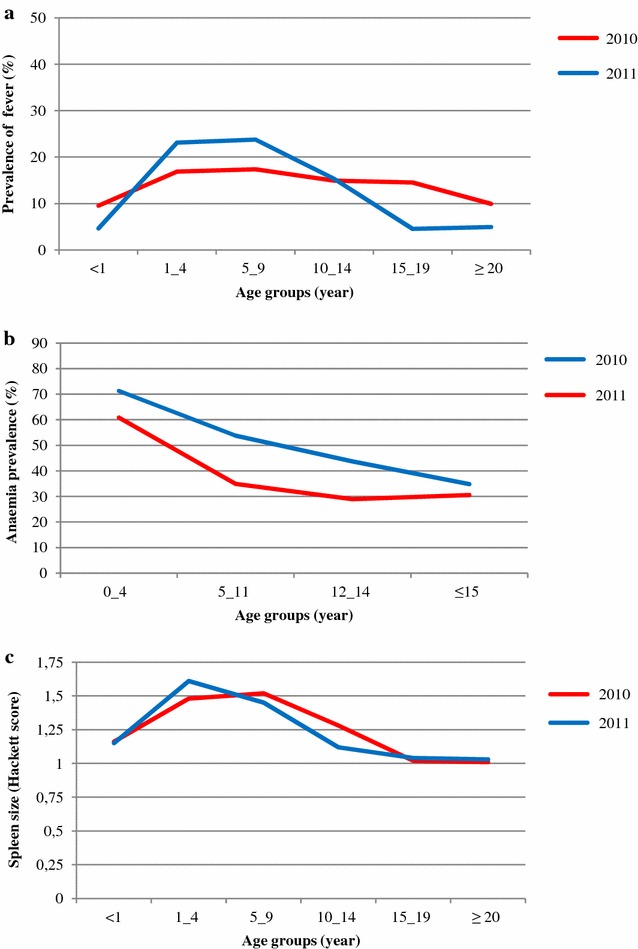
Age-specific prevalence of fever (**a**), anaemia (**b**) and mean spleen size (**c**) of the study population in the Taabo HDSS

Ear temperature measurements were available for 1095 and 1187 of the surveyed individuals in 2010 and 2011, respectively. Fever, as defined by an ear temperature ≥37.5 °C, was found in 13.0 % of the individuals tested with no difference between the two surveys. The occurrence of fever showed an association with area of residency; in 2010, a statistically significantly higher prevalence was found in rural compared to the urban area (*p* < 0.001). With regard to sex, no difference was found in the prevalence of fever (Table 3). Age was associated with fever; children were found with higher prevalence of fever than adults aged 20 years and above (Fig. 2a). Among the febrile individuals, 55.1 % were *Plasmodium*-positive in 2010 and 67.1 % in 2011, whereas among non-febrile individuals, the respective prevalences were 45.0 and 56.8 %. There was a significant association between *Plasmodium* infection and fever (Table 4).

**Table 3:** Prevalence of fever and anaemia among the sample population in the Taabo HDSS, stratified by year of survey

	Fever (%)		Anaemia (%)	
	2010 (N = 1095)	2011 (N = 1187)	2010 (N = 1087)	2011 (N = 1196)
Overall	13.4	13.3	47.6^a^	37.3^b^
Gender				
Male	13.8 (74/538)	13.0 (71/548)	45.0 (241/536)	34.3 (190/554)
Female	13.1 (73/557)	13.6 (87/639)	50.1 (276/551)	39.9 (256/642)
*p*-value	0.821	0.804	0.102	0.053
Residence				
Hamlets	15.4^a^ (29/188)	17.9 (34/190)	45.7 (84/184)	38.2 (73/191)
Villages	15.2^a^ (109/718)	12.9 (110/851)	49.0 (350/714)	37.7 (323/856)
Urban	4.8^b^ (9/189)	9.6 (14/146)	43.9 (83/189)	33.6 (50/149)
*p*-value	<0.001	0.069	0.380	0.600

For each variable, different letters in the same row or column indicate that their difference is significant at level of 0.05

*N* total number of individuals examined

**Table 4:** Relations between *Plasmodium* infection and fever, anaemia and splenomegaly in the Taabo HDSS, stratified by year of survey

Thick film	2010		2011	
	Fever −	Fever +	Fever −	Fever +
Positive	427	81	584	106
Negative	521	66	445	52
Prevalence	45.0 %	55.1 %	56.8 %	67.1 %
OR	1.00	1.49 (1.05–2.12)	1.00	1.55 (1.09–2.22)
	*p*-value = 0.023		*p*-value = 0.014	

Thick film	Anaemia −	Anaemia +	Anaemia −	Anaemia +
Positive	228	279	414	280
Negative	342	238	336	166
Prevalence	40.0 %	54.0 %	55.2 %	62.8 %
OR	1.00	1.75 (1.38–2.23)	1	1.36 (1.07–1.74)
	*p*-value < 0.001		*p*-value = 0.010	

Thick film	Hackett class		Hackett class	
	Class = 1	Class > 1	Class = 1	Class > 1
Positive	405	94	567	118
Negative	529	46	495	24
Prevalence	43.4 %	67.1 %	53.4 %	83.1 %
OR	1.00	2.67 (1.84–3.91)	1.00	4.29 (2.77–6.91)
	*p*-*value* < 0.001		*p*-*value* < 0.001	

*OR* odds ratio

Of the *Plasmodium*-positive individuals, depending on the year of survey, the proportion of fever cases ranged between 15.4 and 15.9 %. Significantly more fever cases were found among heavily infected individuals (parasitaemia ≥10,000 parasites/μl of blood) compared to those with only light or moderate infection (28.9–45.9 versus 13.6–14.7 %; *p* < 0.05).

### Anaemia and relationship with *Plasmodium* infection

Hb measurements were available for 1087 and 1196 individuals in 2010 and 2011, respectively, which allowed estimating the prevalence of age- and sex-specific anaemia. In the 2010 cross-sectional survey, anaemia was determined in 47.6 % of the individuals. One year later, the proportion of anaemic individuals was considerably lower; 37.3 % (*p* < 0.01) (Table 3). In both surveys, rates of anaemia were similar between males and females and place of residency (Table 3). There was a tendency for the proportion of anaemic individuals to decrease with age, as shown in Fig. 2b. Among anaemic individuals, the prevalence of *Plasmodium* infection was 54.0 % in 2010 and 62.8 % in 2011, whereas among non-anaemic individuals, the respective prevalence was 40.0 and 55.2 %. *Plasmodium* infection was significantly more prevalent in people with anaemia than in those without (Table 4).

### Splenomegaly and relationship with *Plasmodium* infection

Table 5 shows the extent of splenomegaly within the surveyed population. Most participants (87.0–88.2 %) had a spleen size of 1 on the Hackett scale. The average Hackett score was 1.23 (SD = 0.67) in 2010 and 1.22 (SD = 0.67) in 2011, with no statistically significant difference between the two surveys.

**Table 5:** Distribution of splenomegaly within the study population in the Taabo HDSS, stratified by year of survey

Spleen size	2010	2011	Total
Hackett class 1	934 (87.0 %)	1062 (88.2 %)	1996 (87.6 %)
Hackett class 2	59 (5.5 %)	39 (3.2 %)	98 (4.3 %)
Hackett class 3	52 (4.8 %)	76 (6.4 %)	128 (5.6 %)
Hackett class 4	27 (2.5 %)	23 (1.9 %)	50 (2.2 %)
Hackett class 5	2 (0.2 %)	4 (0.3 %)	6 (0.3 %)
Total	1074 (100 %)	1204 (100 %)	2278 (100 %)

In the 2010 survey, males had significantly larger spleens than females (Table 6). With regard to participants’ residency, the mean spleen sizes measured in 2010 were similar in hamlets, villages and the urban setting, whilst in 2011, spleen sizes were significantly lower in the urban setting compared to villages and hamlets (*p* = 0.002). There was a clear decline in spleen size with age (Fig. 2c). Enlarged spleen (Hackett class >1) was associated with a higher odds of *Plasmodium* infection (Table 4).

**Table 6:** Mean spleen size of the sample population in the Taabo HDSS, stratified by year of survey

	2010		2011	
	Examined	Spleen size	Examined	Spleen size
Total	1074	1.23	1204	1.22
Male	526	1.28	559	1.26
Female	548	1.18	645	1.20
	*p*-value = 0.031		*p*-value = 0.135	
Hamlets	183	1.21	221	1.29^a^
Villages	703	1.25	837	1.23^a^
Urban	188	1.17	146	1.08^b^
	*p*-value = 0.210		*p*-value = 0.002	

Difference between a and b is significant at level of 0.05

## Discussion

This work presents results from one of the few repeated cross-sectional, population-based malaria prevalence surveys conducted at the district level in a highly malaria-endemic setting of West Africa. The study included all age groups, and hence, not only those who are considered at highest risk of malaria (i.e. children below the age of 5 years and women of child bearing age). Furthermore, the study provides stratified data for the three main residential settings of the Taabo HDSS; namely, hamlets, villages and a small town [9]. High *Plasmodium* infection prevalence rates were recorded in all age groups, ranging from 46.0 to 56.6 %. The two cross-sectional surveys were conducted in the rainy season (June/July), which might, at least partially, explain the high prevalence of infection. Levels of *Plasmodium* infection prevalence observed in the current study were several-fold higher than those reported in a lowland village of Tanzania (13 %) [20], but slightly lower than in previous studies undertaken in central and northern Ghana (58–61 %) [21, 22]. In the current study in south-central Côte d’Ivoire, the prevalence of *Plasmodium* infection was somewhat higher in the 2011 survey compared to the preceding year. It will be important to longitudinally monitor *Plasmodium* infection prevalence in the general population of Côte d’Ivoire as control and elimination efforts are being escalated. The Taabo HDSS offers a unique platform in this regard and will allow for cross-country comparisons [23].

There is evidence that the age-pattern of *Plasmodium* infection is dependent on the transmission intensity [24]. In areas of low transmission, the risk of infection is similar in all age groups [25, 26]. Conversely, in highly endemic areas, it is well established that infection rates are higher among children below the age of 5 years, compared to school-aged children, adolescents and adults [24, 27]. In the current study, highest infection rates were observed in children, particularly those aged 1–9 years. The relatively low prevalence observed in infants (age group <1 year) might be explained by the presence of maternal antibodies against malarial antigens, particularly in infants younger than 6 months [28]. For all children, continued exposure to infection may contribute to the development of partial immunity. At the time of this study, based on *Plasmodium* infection rates in children, the district of Taabo can be classified as being hyperendemic for malaria [19].

Males showed significantly higher infection rates than females. This result is supported by previous studies conducted elsewhere in Africa [29], Asia [30, 31] and Latin America [32]. This observation might be explained by gender-specific behaviours. It has been suggested that males tend to be more active with outdoors in the evening than females, thus at higher risk of exposure to mosquito bites that transmit malaria [33]. Another plausible explanation is the fact that females are more likely to use preventive measures against mosquito bites (e.g. long clothes, sleeping under LLINs). It could also be that females use prompt treatment once signs and symptoms of malaria are suspected. Further investigations to deepen the understanding of gender-related risk factors for malaria transmission are warranted and the Taabo HDSS provides a platform for this endeavour.

Preceding studies documented higher *Plasmodium* infection prevalence rates in rural compared to urban areas [34–36]. In the current setting, the prevalence of *Plasmodium* infection was similar in hamlets and Taabo-Cité, the only urban area of the Taabo HDSS. This finding indicates that, in the urban setting, ecological conditions are suitable for malaria transmission. A possible explanation is that Taabo-Cité is located in close proximity to Lake Taabo. Surprisingly though, among the rural areas studied, *Plasmodium* infection was more prevalent in villages than in the less developed hamlets. This observation suggests different risk factors for malaria transmission in the two types of rural settings. Interestingly, different observations have been reported for the western part of Côte d’Ivoire; among children below the age of 15 years, *Plasmodium* infection rates were similar in villages and hamlets [37].

In the current study, *P. falciparum* was the predominant malaria species, while only a few individuals were found positive for *P. malariae* and *P. ovale*. These findings are consistent with epidemiological studies undertaken in 2001 in a single village of the study area [38] and elsewhere in Côte d’Ivoire [39–41]. In sub-Saharan Africa, *P. malariae* and *P. ovale* can reach high prevalence rates [42], but *P. falciparum* is the predominant species [28, 43].

Most infections were of low to moderate parasitaemia with no statistically significant difference between males and females. Although a marked variation in the level of parasitaemia was recorded between villages and the urban setting in 2011, a clear age-pattern was commonly observed in both cross-sectional surveys. This is a reflection of the degree of immunity to the disease, which is known to be low among children, but increases markedly over the time as a result of continuous exposure to *Plasmodium* infection. In the current study, parasitaemia peaked in children ≤9 years of age, and thereafter showed a substantial decline, suggesting that an effective immune response is acquired in early childhood. This may explain that most infections were asymptomatic. Indeed, only a relatively small number of infected persons were found febrile in the community (15.4–21.9 %). However, it is important to note that significant proportions of febrile individuals were diagnosed as having a *Plasmodium* infection (55.1–67.1 %).

Even though fever was positively associated with *Plasmodium* infection, a considerable proportion of fever cases (32.9–44.9 %) showed no *Plasmodium* infection. This observation raises the question of whether febrile patients should be treated empirically for malaria. In the current study area, presumptive treatment of malaria is common, even in health facilities. As this practice results in overtreatment and might lead to the development of anti-malarial drug resistance, there is a need for RDTs and treatment of only those cases found positive [8, 44].

The prevalence of anaemia was high within the surveyed population (37.3–47.6 %). Not surprisingly, children were at highest risk, which is in line with previous studies conducted in Kenya [45], Nigeria [46] and Cameroon [47]. The association between *Plasmodium* infection and anaemia supports the finding that malaria plays a major role in the occurrence of anaemia in tropical areas [48–50]. Nevertheless, other causes cannot be excluded. In recent surveys conducted in the study area, it was shown that, depending on age groups, *Schistosomahaematobium* infection, inflammation, cellular iron deficiency and chronic malnutrition must be considered as important contributors to anaemia [15, 51]. This may explain why high prevalence of anaemia was recorded within the populations. However, a decreasing trend in anaemia was observed over time. Since 2009, several rounds of deworming were administered, which is a probable explanation for the decrease of the proportion of anaemic persons [9].

The proportion of people with enlarged spleen might serve as an indicator for the intensity of malaria transmission [52]. Evidence from the current study suggests that the high proportion of enlarged spleens in the surveyed population might be an indication of latent and chronic infections. Not surprisingly, the data revealed an association between *Plasmodium* infection and splenomegaly with spleen rates showing similar trends as parasite rates. This is expected for malaria-endemic countries, where transmission is intense and perennial [53–55]. Although malaria is a leading cause of splenomegaly, enlarged spleen is known to be multifactorial [56]. For example, schistosomiasis and other helminthiases should be considered as potential additional causes of splenomegaly. Indeed, in a context of persistent high malaria transmission, the observed decline in the mean spleen size and anaemia from 2010 to 2011 might be explained by the regular deworming targetting schistosomiasis and soil-transmitted helminthiasis.

## Conclusion

This study provides important information on age-, sex- and residency-specific *Plasmodium* infection rates, parasitaemia and anaemia in the Taabo HDSS where malaria remains the main cause of death. Indeed, the public health significance of malaria calls for concerted efforts to control this mosquito-borne disease. The prevalence of infection was particularly elevated in children below 10 years of age. Males and village residents were at higher risk than females and people living in hamlets or Taabo-Cité. The association between *Plasmodium* infection and fever, anaemia and splenomegaly, which is a typical feature of heavily endemic areas, demonstrates the severity of the disease. There is, therefore, a pressing need to scale-up control measures in order to reduce the burden of malaria in the Taabo HDSS and Côte d’Ivoire more generally. Further investigations on malaria risk factors will enable a comprehensive overview of the heterogeneities observed within the residence settings and the two genders to come up with setting-specific control interventions.

## Abbreviations

ACT: artemisinin-based combination therapy
CSRS: Centre Suisse de Recherches Scientifiques en Côte d’Ivoire
HDSS: health and demographic surveillance system
Hb: haemoglobin
LLIN: long-lasting insecticidal net
OR: odds ratio
RDT: rapid diagnostic test
SD: standard deviation
WBC: white blood cell
WHO: World Health Organization
